# Bilateral acute retinal necrosis in a patient with multiple sclerosis on natalizumab

**DOI:** 10.1186/s12348-016-0095-y

**Published:** 2016-07-20

**Authors:** Arjun B. Sood, Gokul Kumar, Joshua Robinson

**Affiliations:** Department of Ophthalmology, Emory University, Atlanta, GA USA; Emory Eye Center, Atlanta, GA USA

**Keywords:** Herpes virus, Immunosuppression, Multiple sclerosis, Natalizumab, Retinal necrosis

## Abstract

**Purpose:**

The purpose of this study is to report a case of bilateral acute retinal necrosis in a patient with multiple sclerosis treated with natalizumab.

**Methods:**

This study is a case report and literature review.

**Results:**

A 34-year-old Caucasian female with multiple sclerosis presented with 1 week of blurry vision in both eyes during treatment with natalizumab. Clinical examination revealed bilateral acute retinal necrosis. The patient was treated with systemic intravenous acyclovir and intravitreal injections foscarnet and ganciclovir. Natalizumab therapy was also discontinued.

**Conclusions:**

Natalizumab is a potent immunosuppressive agent used in relapsing remitting multiple sclerosis and Crohn’s disease. The use of this medication is commonly associated with opportunistic infections in the CNS. In rare cases, ocular opportunistic infections may occur and can lead to significant visual impairment and blindness. Neurologists and ophthalmologists should be aware of this potential complication.

Acute retinal necrosis (ARN) is a rare infectious uveitis that is caused by members of the herpes family including varicella zoster virus (VZV), herpes simplex virus (HSV), cytomegalovirus (CMV), and less frequently Epstein-Barr virus (EBV) [1]. The syndrome is characterized by necrotizing retinitis that can lead to severe visual impairment and blindness, even despite prompt diagnosis and management [2, 3]. Here, we present a unique case of bilateral ARN in patient with multiple sclerosis on natalizumab immunosuppression.

## Case report

**Fig. 1:**
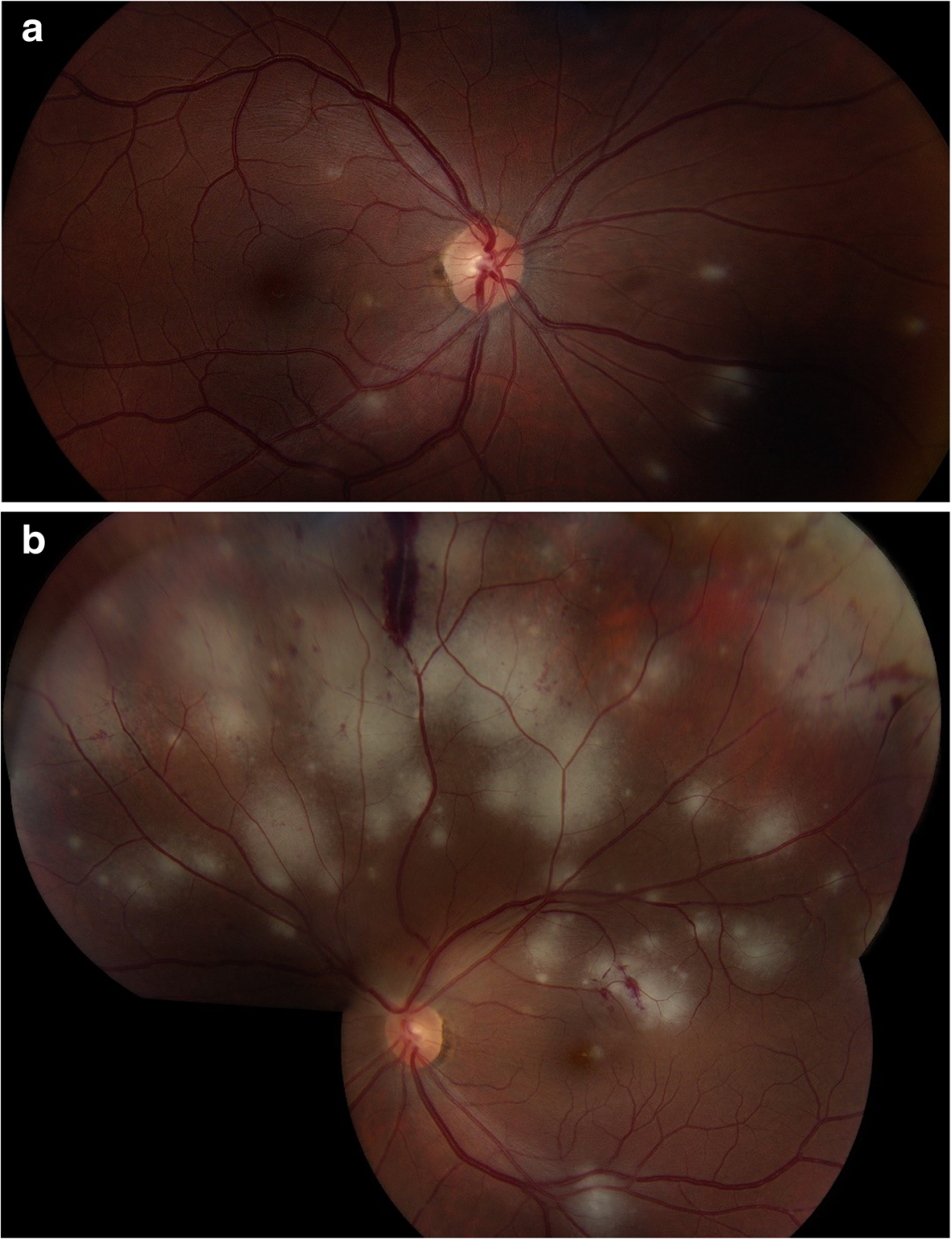
Funduscopic examination showed areas of retinal whitening in the macula and periphery in the right eye **(a)** and left eye **(b)** with more confluent areas of necrosis in the left eye associated with retinal hemorrhage

A 34-year-old Caucasian female presented to the Emory Eye Center in March 2015 with complaints of blurry vision in both eyes for the past 1 week. The patient’s past medical history is notable for multiple sclerosis (MS) diagnosed in 2011 after an episode of bilateral optic neuritis and gait instability. Her MS was initially managed with interferon beta-1a in 2011, but due to recurrent flares, she was transitioned to natalizumab in 2012. She had received monthly infusions of natalizumab with the last infusion 3 weeks prior to presentation. Visual acuity was 20/25 right and 20/50 eccentrically left eye. The pupils were equal and reactive without a relative afferent pupillary defect. Intraocular pressures were 14 in both eyes. Extraocular motility was full and non-painful in both eyes. Anterior segment exam was notable for trace cell in the right eye and 2 + cell in the left eye. Funduscopic examination showed areas of retinal whitening in the macula and periphery in both eyes with more confluent areas of necrosis in the left eye associated with retinal hemorrhage (Fig. 1a, b).

The patient’s clinical presentation was consistent with bilateral acute retinal necrosis, and she was admitted for further evaluation and management. A diagnostic anterior chamber (AC) paracentesis was performed, and the sample was tested for VZV, HSV, CMV, and toxoplasmosis PCR. The patient was given intravitreal injections of foscarnet 2.4 mg/0.1 cm^3^ and ganciclovir 2 mg/0.1 cm^3^ in both eyes and administered intravenous acyclovir. The diagnostic AC paracentesis was positive for VZV, and systemic work-up was unrevealing for HIV. Magnetic resonance angiography was negative for evidence of CNS vasculitis. After consultation with neurology, natalizumab was discontinued. Over the next several months, the patient was given multiple bilateral intravitreal injections and continued on systemic oral valacyclovir 1 g three times a day. The infection in the right eye resolved, and she maintained good vision at 20/30 at the last follow-up. However, despite aggressive therapy, the retinitis in the left eye rapidly spread throughout the macula and went on to involve the optic nerve with a drop in vision to hand motion. The patient later developed a macular-sparing combined tractional and rhegmatogenous retinal detachment in the left eye. Four months after presentation, the patient underwent scleral buckling and pars plana vitrectomy with silicon oil and successful reattachment of the retina. Visual acuity in the left eye at the last follow-up was hand motion, limited by macular and optic atrophy. The patient remains off natalizumab and is taking oral valacyclovir 1 g daily.

## Discussion

The case presented herein represents an atypical presentation of acute retinal necrosis. ARN is an infectious uveitis caused by members of the herpes family. The syndrome is characterized by necrotizing retinitis, vitritis, occlusive vasculitis, and rapid progression in the absence of antiviral therapy [3]. ARN is a rare ocular condition and the exact incidence in the USA is unknown; however, evidence from the UK in 2007 estimates an incidence of one case per 1.6 to two million population per year [4].

Risk factors for the development of ARN include recent herpes virus infection, genetic predisposition, and history of immunosuppression. Classically, corticosteroids have been associated with the development of ARN [5]. More recently, natalizumab has been implicated in the development of herpetic retinal necrosis [6–8]. Natalizumab is a humanized monoclonal antibody used in the treatment of relapsing and remitting MS and Crohn’s disease [9].

Natalizumab binds to the alpha-4 subunit of integrins expressed on leukocytes and interferes with their ability to adhere to endothelial cells and migrate into the central nervous system and gastrointestinal tract. In patients with MS, natalizumab decreases the CD4+/CD8+ ratio in the CSF compared to peripheral blood resulting in impaired immune surveillance [9, 10]. Patients on natalizumab are at risk for opportunistic infections. The most feared infection is progressive multifocal leukoencephalopathy caused by the JC virus. Recent reports by the US Food and Drug Administration (FDA) and others suggest a growing association with herpes-related infections in the CNS and eye [10].

Table 1 illustrates the three previously reported cases of herpetic retinal necrosis in patients receiving natalizumab [6–8]. All patients discontinued natalizumab, and one case utilized plasma exchange to accelerate natalizumab clearance. All patients received systemic antivirals.

**Table 1:** Clinical characteristics and visual outcomes in patients with herpetic retinal necrosis associated with natalizumab

Publication	Age (years)	Sex	Laterality	
Kobeleva et al.	49	M	Bilateral	
Saraiva	51	M	Unilateral (right eye)	
Van Tassel et al.	54	F	Unilateral (left eye)	
Current study	34	F	Bilateral	
Publication	Initial VA	Treatment/clinical course	Length of follow-up	VA at the last follow-up
Kobeleva et al.	Not reported	CNS vasculitis and necrotizing retinitis treated with combination of IV acyclovir, high-dose IV methylprednisone and 5 cycles of plasma exchange therapy to accelerate natalizumab clearance.	4 months	“Almost completely blind.”
		Cyclophosphamide was given after steroids and plasma exchange.		
Saraiva	20/30 OD	Natalizumab was discontinued. Oral valacyclovir 1 g TID for 3 months. Oral prednisone initiated 4 days after starting valacyclovir. Propophylatic laser retinal photocoagulation performed 14 days after initiation of antiviral therapy. One month after discontinuing antiviral therapy, the patient developed immune reconstitution inflammatory syndrome. Systemic steroids resolved inflammation.	5 months	20/30 OD
Van Tassel et al.	20/125 OS	Natalizumab was discontinued. IV and intravitreal antivirals were administered. Complicated by late retinal detachment.	2 months	20/125 OS
Current study	20/25 OD, 20/50 OS	Natalizumab was discontinued. IV and intravitreal antivirals were administered. Complicated by late retinal detachment and optic atrophy.	11 months	20/30 OD, HM OS

Our patient was treated with systemic intravenous and oral antivirals and intravitreal injections of foscarnet (2.4 mg/0.1 cm^3^) and ganciclovir (2 mg/0.1 cm^3^). Despite appropriate therapy, our patient developed a combined tractional and rhegmatogenous retinal detachment in the left eye. She underwent scleral buckling and pars plana vitrectomy with silicon oil with successful reattachment of the retina. However, vision remains hand motion at the last follow-up due to macular and optic nerve involvement of the necrotizing retinitis. The right eye was successfully treated and vision remained 20/30.

## Conclusions

Acute retinal necrosis is a rare infectious uveitis that can lead to significant visual impairment and blindness despite prompt diagnosis and management. Patients with history of natalizumab immunosuppression must be treated aggressively. We recommend discontinuation of natalizumab and intensive treatment with antivirals. As with other cases of ARN, there is a high risk of retinal detachment and severe vision loss. Consideration should be given to plasma exchange therapy to accelerate natalizumab clearance but should be done only in coordination with neurology and infectious disease teams.
